# Civil society involvement in harm reduction drug policy: reflections on the past, expectations for the future

**DOI:** 10.1186/s12954-020-00426-8

**Published:** 2021-02-10

**Authors:** Aileen O’Gorman, Eberhard Schatz

**Affiliations:** 1 School of Education and Social Sciences, University of the West of Scotland, Paisley, UK; 2 Correlation - European Harm Reduction Network, Droogbak 1d, Amsterdam, 1013 GE The Netherlands

## Abstract

**Background:**

A range of civil society organisations (CSOs) such as drug user groups, non-governmental/third sector organisations and networks of existing organisations, seek to shape the development of drugs policy at national and international levels. However, their capacity to do so is shaped by the contexts in which they operate nationally and internationally. The aim of this paper is to explore the lived experience of civil society participation in these contexts, both from the perspective of CSOs engaged in harm reduction advocacy, and the institutions they engage with, in order to inform future policy development.

**Methods:**

This paper is based on the presentations and discussions from a workshop on ‘Civil Society Involvement in Drug Policy hosted by the Correlation - European Harm Reduction Network at the International Society for the Study of Drugs Policy (ISSDP) annual conference in Paris, 2019. In the aftermath of the workshop, the authors analysed the papers and discussions and identified the key themes arising to inform CSI in developing future harm reduction policy and practice.

**Results:**

Civil society involvement (CSI) in policy decision-making and implementation is acknowledged as an important benefit to representative democracy. Yet, the accounts of CSOs demonstrate the challenges they experience in seeking to shape the contested field of drug policy. Negotiating the complex workings of political institutions, often in adversarial and heavily bureaucratic environments, proved difficult. Nonetheless, an increase in structures which formalised and resourced CSI enabled more meaningful participation at different levels and at different stages of policy making.

**Conclusions:**

Civil society spaces are colonised by a broad range of civil society actors lobbying from different ideological standpoints including those advocating for a ‘drug free world’ and those advocating for harm reduction. In these competitive arena, it may be difficult for harm reduction orientated CSOs to influence the policy process. However, the current COVID-19 public health crisis clearly demonstrates the benefits of partnership between CSOs and political institutions to address the harm reduction needs of people who use drugs. The lessons drawn from our workshop serve to inform all partners on this pathway.

## Background

In recent decades, a range of civil society organisations (CSOs)^1^ (such as drug user groups, non-governmental or third sector organisations, and networks of existing organisations) have sought to shape the development of drugs policy at national and international levels. These CSOs engage in peer, professional, and public policy advocacy and seek to effect change mainly through legislation, resource allocation, and service provision [9].

Civil society involvement in drug, and other, policy making has been enabled by the expansion of ‘democratic spaces’ for civil society participation at national and supranational level [3, 6]. By participating in such spaces (e.g. by writing submissions, or contributing to government committees and consultative fora) and seeking to influence the formal policy making process, many CSOs have shifted focus from their traditional ‘outsider’ activist role to adopt an ‘insider’ strategy [2] in the belief that social change occurs through politics [11].

However, the capacity for civil society to influence harm reduction discourses and drug policies from the inside is shaped by the contexts in which they operate. At a national level, the level of ‘enabling environment’ for civil society is key [4]. So too are the prevailing views regarding drug use and harm reduction, the extent and range of public health services to address drug-related harms; and the local level of drug regulation and law enforcement [9]. At a supra national level, there are spaces with varying levels of scope for influencing drug policy. For example, the Vienna NGO committee (VNGOC) facilitates CSO access to the United Nations Office of Drugs and Crime and the Commission on Narcotic Drugs (CND); and the EU Civil Society Forum on Drugs provides a platform for dialogue and interaction with the European Commission and for feeding grassroots experience, expertise, and recommendations into European drug strategies and action plans.

CSO participation in policy development, setting and implementation in these spaces is regarded as mutually beneficial to all stakeholders by facilitating dialogues between civil society (seeking to influence policy, and/or achieve social justice reform) and national, EU and transnational governance bodies (seeking to develop more inclusive and grounded policies [7, 8]. However, there are limitations to the level and scope for influence in the mainly consultative fora civil society is invited to join in the drugs field. The agenda of these fora are limited mainly to treatment and demand reduction issues and are shaped by the paradigm of prohibition enshrined in the international drug control conventions. Furthermore, these spaces are colonised by a broad range of civil society actors lobbying from different ideological standpoints such as those campaigning for a ‘drug free world’ and those advocating for harm reduction. In these competitive arena it may be difficult for harm reduction orientated CSOs to establish power and influence over the policy process.

In addition, levels of participation in these fora varies—ranging from minimalist and tokenistic forms, such as providing information and rudimentary consultation, to direct involvement in the decision-making processes (see Arnstein [1] ‘Ladder of Participation’). By Arnstein’s measure, real participation means the power to make decisions. However, more nuanced models of participation in decision-making processes have been developed that more accurately reflect the *realpolitik* of engagement where CSOs participation is at the request of national and international institutions [5].

The extent to which CSOs can influence power and engage in meaningful participation in an unequal setting is an ongoing concern for CSOs [13]. So too are their efforts to assert legitimacy to act on behalf of their ‘constituents’ and countermand the politicians and public servants who critique CSOs for being unaccountable elites [14, 15].

The aim of this paper is to explore the lived experience of civil society participation at the national and international levels from the perspectives of both CSOs engaged in harm reduction advocacy, and the institutions they engage with.

## Methods

This paper is based on the presentations and discussions from a half-day workshop on ‘Civil Society Involvement in Drug Policy- what can we learn from each other from a research, policy and civil society perspective.’ The workshop was hosted by the Correlation - European Harm Reduction Network at the 16th annual conference of the International Society for the Study of Drugs Policy (ISSDP) in Paris, 2019.

The workshop critically analysed the role of civil society involvement (CSI) in harm reduction drug policy and presented examples of CSI at UN, EU and national level. Key themes addressed at the workshop included: the importance of CSI; achievements to date; successes and challenges; and how CSI can be improved for the future.

The invited speakers represented a broad range of stakeholders involved in CSI processes (see Acknowledgements for full details). These included civil society NGOs, networks and groups, and research and policy agencies. After the keynote speakers presentations, the workshop participants (PWUDs, academics, policy makers, and CSO members) discussed the presentations and identified recommendations for a shared way forward.

In the aftermath of the workshop, the two authors analysed the papers and discussions and identified the key themes arising from the workshop in order to inform CSI in developing future harm reduction policy and practice.

## Findings

### Perspective of CSOs: drug users groups

From the perspective of drug user organisations, participating in policy fora provides an opportunity to inform harm reduction policy and practice with lived experience. However, there were considerable limitations and drawbacks to their involvement also, not least because the quality of participation permitted varied substantially from context to context.

Mat Southwell (European Network of People Who Use Drugs, UNODC Civil Society Group on Drug Use and HIV Secretariat) reflected on his experience representing people who use drugs in both the UK and the UN. Southwell recounted the heady days of drug user activism in the UK in the 1980s when they were at the forefront of the grassroots harm reduction movement responding to HIV and AIDS in their community. As activists, they mobilised to provide needle and syringe programmes often before these had been legally sanctioned by the state. Later, in the 1990s, drug user groups involved in the dance ‘rave’ scene were influential in providing alternative harm reduction perspectives on drug use in recreational settings (primarily Ecstasy), as well as a critique of prohibition.

In the 2010s, Moving from ‘outsider’ activism to ‘insider’ participation in consultative and policy fora proved to be a challenging experience. The focus of the leadership of drug user groups switched from activism to high-level negotiations, network building, and the establishment of representative structures often in adversarial and heavily bureaucratic environments. Southwell’s account highlighted some key issues. First, what Southwell identified as a ‘clash of expectations’ between the user groups and state agencies, with the latter seeking to frame drug user participation in terms of a consumer relationship between agencies and people in treatment, whereas the drug user groups (informed by a human rights analysis and a broad constituency of people who used drugs, not just those seeking treatment), sought an open dialogue, and a more meaningful partnership with the Government.

The drug user groups found also a differentiated level of commitment to partnership among staff in government agencies: some were committed to supporting meaningful participation and others were either ideologically opposed to CSI, or opposed because of interpersonal and inter-departmental rivalries with those in favour. Overall, they found commitments being made but not always implemented, and drug user representation systems being bypassed. A change in government and/or shift in strategy led to abandoned initiatives, key allies moved elsewhere, and new policy initiatives and structures implemented. As a result, members became frustrated at the limited impact at the grassroots level, trust in the leadership was undermined, individual leaders became overstretched and demoralized which impacted on their drug using behaviour.

In contrast, the drug user groups experience at European and international level was a more positive experience. Initially, INPUD had been appointed as a member of the UK Delegation to CND. Even though this appointment was terminated after two years due to a change in Government and strategic approach, INPUD’s participation in CND was a key learning process for the network as it enabled them to develop their political skills in navigating complex international institutions. Mentoring support provided by sympathetic public servants and more experienced civil society activists eased their transition from outsiders to insiders. This experience, and the key relationships they formed there, enabled INPUD to secure a place on the UNAIDS Programme Coordinating Board (PCB). Here, formal support and a funded secretariat for the NGO representatives enabled more strategic participation. For example, their contribution to a civil society advocacy intervention led to the creation of the UNODC Civil Society Group on Drug Use and HIV.

More recent developments, such as the UN adopting the GIPA principles^2^ have enabled more meaningful participation. For INPUD and the other global key population networks, this means they have specific a line of communication and engagement in the system, and a recognised and valued contribution to a variety of civil society participation mechanisms.

### Perspectives of CSOs: networks and service providers

The European Civil Society Forum on Drugs (CSFD), comprising of 45 non-governmental organisations (NGOs), was established by the European Commission to act as a consultative expert group to provide views, inputs and advice on European Drug Policy (such as its five year drug strategy and related Drug Action Plan) and to provide a common EU position on drug policy at international level.

Laurene Collard, Chair of the CSFD, noted how their consultative body status enables CSOs to access key stakeholders, and work on joint publications, written contributions and statements. However, the Forum represents a diverse group of CSOs from different national contexts with a broad range of views on drugs and addictions. Coordinating such a diverse organisation proved challenging when it came to publishing common positions on specific issues such as harm reduction. Decision-making in this context requires a strong dialogue among the CSFD members. This process was eased by the establishment of a core group of elected coordinators of working groups. These groups provide a legitimate framework for discussions and to identify the main common position.

As with other CSOs, the CSFD found that adequate resources are key for participation. Initially, the Commission provided funding for the CSFD’s annual plenary meeting only. Now, activities are resourced by a grant which funds five designated NGOs to undertake the working groups’ activities and communication.However, the renewal of their funding is at a different timeline than the renewal of the CSFD’s mandate. These anomalies are a constant challenge to be faced with EU policy bodies working with timelines, priorities and operating rules different to those of the NGOs.

Another challenge faced by the CSFD was its attempt to access policy making spaces outside their invited space in the Commission. For example, in addition to working with the European Commission (which formalises European policies), the CSFD sought access to the main European Council (where policies are agreed by member states) and its Horizontal Working Party on Drugs (HDG) which is responsible for leading and managing the Council's work on drugs. This proved difficult for the CSFD which Collard attributed to different ways of working; different timelines; different agendas; and different priorities rather than barriers erected by the HDG itself.

The Correlation - European Harm Reduction Network (C-EHRN) is an active and longstanding member of the CSFD. C-EHRN is a network of over 200 CSOs based in Europe with a specific remit to advocate for harm reduction policies and practice. Eberhard Schatz reported on C-EHRN’s experience connecting harm reduction services, grassroots organisations, research institutes, and health facilities from all over Europe, as well as cooperating with partners and policy makers at local, national and European levels.

From C-EHRN’s perspective, the inclusion of civil society in the development and implementation of drug policies provides added context to policy considerations; gives policy makers access to a greater range of insights and information, and can support the popular legitimacy of policy actions. In short, a structured and formal civil society involvement in policy arenas can better equip states to plan, implement and measure policy initiatives, thus directly contributing to national and EU drugs strategy objectives.

Despite these positive outcomes, the reality of CSO participation often did not meet with expectations. Schatz outlined the key barriers to effective CSI that C-EHRN had identified, particularly at the national level. These were:

- the lack of structural and formalised consultation;
- the mismatch between policy and practice;
- the lack of knowledge among CSOs on how to get involved in policy-making; and
- the lack of awareness among policy makers on the positive effects of CSI.

As a result of this analysis, a group of CSOs embarked on a collaborative project—the European Civil Society Involvement Project (CSIDP)—to assess civil society involvement at European and national level; to stimulate inclusion of CSO’s in drug policy decision making; and, inform how good quality CSI could be best achieved by both states and CSOs (see https://csidp.eu/).

Drawing from the Council of Europe ‘Code of Good Practice for Civil Participation in the Decision-Making Process’ [5], the CSIDP group identified the multiple spaces where there was scope for CSI at different stages of the policy cycle: during agenda setting, policy formulation, decision-making, implementation, evaluation or possibly the re-formulation of policies. They also noted the ascending scale of different levels of CSI: Information—Consultation—Dialogue—Partnership.

The group’s analysis resulted in the development of a Road Map and other publications for both policy makers and civil society organisations to improve CSI and make it more meaningful and successful (see [7, 8, 12]. For policy makers, they recommended that governments at all levels should invest adequate time and (financial) resources in building robust legal, policy and institutional frameworks, and evaluating their own performance in engaging CSOs in policy- and decision-making processes. Commitment and leadership by politicians and senior public officials were noted as key ingredients for CSI, as Southwell had noted also. CSIDP advised CSOs to be ready and able to fulfil a proactive and leading role in this process, to build leadership, strategic management and technical capacity, and to explore opportunities for funding to enable participation. They concluded that although CSI is a ‘hot’ topic at European level, often it is not a high priority at national level in many countries.

A CSI case study presented by Marcus Keane from the Ana Liffey Drug Project in Ireland provided a good example of the challenges experienced by CSOs promoting harm reduction at a national level. This presentation illustrated the complex nature of translating evidence (for the establishment of a Safe Injecting Facility (SIF) in Dublin) into policy and practice. Their experience demonstrated the need to engage in all levels of the decision-making process (as Schwartz had outlined previously), namely agenda setting; drafting; decision-making; implementation; monitoring and reformulation. For example, CSOs in Ireland had played a lead role in setting the agenda for a SIF by advocating, drafting legislation, winning the hearts and minds of politicians and public opinion. Yet, in the end, the campaign was halted by planning regulations and the requirement that planning permission had to be obtained for the facility. As a result, the implementation of the policy became stuck in a cumbersome planning process objected by state, commercial, and public interests and is unlikely to be resolved for some time.

Keane noted from this experience that, ‘the state is not a unitary actor—it is a large and often contradictory body of largely separate entities, who are often competing with each other for resources, or because their remits are different.’ As noted by Southwell earlier, CSOs have found that levels of support for an initiative can vary within and between different arms of the state and that the support of one agency does not mean the initiative is supported by others. For example, though the SIF was listed in the Programme for Government in Ireland, and the state Health Service Executive (HSE) conducted the tender process for its operation, another branch of the same state agency (the HSE) lodged an objection to the planning application for the service. This case study demonstrates the complexity of the policy process, and the inherent conflict, competition and rivalries at play in the workings of state institutions that mitigate against evidence-based harm reduction policies being implemented.

### Perspective of a European inter-governmental group (The Pompidou Group)

From the perspective of the Pompidou Group (the Council of Europe’s inter-governmental drug policy cooperation platform)^3^, civil society organisations (CSOs) bring knowledge and independent expertise to the process of decision making. This has led governments at all levels, from local and regional to national, as well as international institutions, to draw on the relevant experience and competence of NGOs to assist in policy development and implementation in drug and other policy.

The Pompidou Group (PG) has been a long time champion of civil society participation as an important part of the democratic process [10]. The Group sees the legitimacy of civil society participation flowing from the European Convention of Human Rights (ECHR) which guarantees the freedom of expression (Art. 10) and the freedom of assembly and association (Art. 11). Following on from these freedoms citizens have the right to make their political opinions and views known and to form, support and join political parties and social movements. In this sense, the wide variety of CSOs representing the diversity of society are complementary to representative democracy and address concerns about the alienation of citizens from the political processes, and the democratic deficit.

Thomas Kattau from the PG noted the benefits and added value of civil society input to the policy planning and implementation process. CSOs are seen to enjoy the trust of their members to represent their interests thereby providing crucial input into policy development. From the perspective of policy makers, this can enhance the legitimacy, quality, understanding and longer term applicability of a policy initiative. Collaborative action between civil society and public authorities leads to more dynamic, efficient and effective policy development and implementation. In the case of drug policy, cross-cutting or network-based civil society actors can often overcome sectorial barriers much easier than the public administrations.

Similar to Schatz, Kattau noted the four gradual levels of civil society participation from least to most participative (information; consultation; dialogue; and partnership) that may be applied at any step in the policy-making process (agenda setting, drafting etc.). Though, as the Dublin case study illustrated these stages tend to be relevant at certain points in the process.

From the Pompidou perspective, there are some limitations to CSI in that government and public institutions have different roles and responsibilities than CSOs and often also different aims and objectives. In addition, their management, administration and resource mobilisation differ significantly. Levels of cooperation are also different at national, regional and local level. Overall, these form ‘compatibility challenges’ that should be recognised and addressed by both sides.

Security sensitive dimensions to drug policy, such as law enforcement, criminal justice systems and customs, are frequently cited as limitations to cooperation with CSOs in these areas. Kattau noted that though these are justifiable and valid concerns they can be used as a barrier for cooperation with civil society actors. Experiences in the international sphere has shown that cooperation with non-government actors can be feasible and possible even in security sensitive areas.

### Perspective of a European agency (The EMCDDA)

The European Monitoring Centre for Drugs and Drug Addiction (EMCDDA) was established to provide the EU and its Member States with factual, objective, reliable and comparable information at European level concerning drugs and drug addiction and their consequences. Nicola Singleton from the EMCDDA noted that though the need for evidence-based policies and practice is widely-recognised in Europe and internationally this can be challenging in the drugs field where evidence can be limited, is constantly evolving, and is context dependent. In addition, evidence may be interpreted in different ways, and is often disputed.

From the EMCDDA perspective, Europe is diverse both in terms of the nature and extent of drug problems, but also in culture, socio-economic circumstances, and administrative structures, and evidence needs to take account of this variability. There are many different stakeholders with an interest in increasing the use of evidence in drug policy and, as resources are scarce, collaboration to make best use of the particular strengths of different groups and avoid duplication is important to maximise impact.

Civil Society is equally diverse both in terms of its composition and also in terms of how and to what extent different groups are involved in drug policy. In their work, the EMCDDA engages with Civil Society in a number of different ways for a variety of purposes. For example, as:

- customers (e.g. an important target group for the EMCDDA Best Practice Portal and publications);
- advisers (e.g. CSO representatives may be members of advisory groups);
- service and data providers (such as drug checking and drug consumption rooms); and
- researchers (e.g. commissioned to conduct studies and reports).

These relationships lead in turn to wider opportunities for collaboration between the EMCDDA and CSOs. For example, the collaboration with Correlation - European Harm Reduction Network (C-EHRN) in identifying activities to promote HCV testing and treatment for people who inject drugs, or the interaction with civil society at policy level within the EU Civil Society Forum on Drugs and on HIV.

For the EMCDDA, CSOs represent important perspectives and are key stakeholders. However, for the agency they are but one of many and the diversity and number of organisations representing different groups poses a challenge. Working with representative groups, such as C-EHRN, the European Network of People who use Drugs, and the Civil Society Fora on Drugs and on HIV, viral hepatitis and TB, is valuable in addressing this issue but the agency may also need to consider which perspectives are most important in any particular situation, and also to try and ensure that they are inclusive of a variety of groups from across the EU.

Since both EMCDDA and CSOs have limited resources there can be mutual benefit from co-operation and collaboration, avoiding duplication and creating synergies. However, as noted similarly by the Pompidou Group representative, the EMCDDA and Civil Society have different roles and mandates. Working together, recognising shared objectives and respecting their different skills and roles will be key to making activities more efficient and improving the use of evidence in drug policy for greater impact.

## Discussion and recommendations

Civil society involvement in policy decision-making and implementation is widely acknowledged as an important benefit to representative democracy. In the field of drug policy, a diverse range of civil society stakeholders bring a variety of experience, knowledge and perspectives to the drug policy debate based on peer, professional and public policy expertise.

In the ISSDP workshop, and civil society literature, two recurring concerns/issues shape the nature and extent of civil society involvement: participation and legitimacy.

For drug user groups, activist organisations, and CSOs operating from a community development ethos, both the process (how things are done) as well as outcomes of civil society involvement (what is achieved) are important. For example, the adoption of the GIPA principles acknowledges the right of affected communities to participate in decision-making processes that affect their lives—‘nothing about us without us’. This approach facilitates a more meaningful level of participation for people who use drugs and the broader professional and public policy CSOs who are involved in all aspects of the policy making process from the planning of agendas, meetings, consultations, the development of guidelines, and policy development.

The Council of Europe (2007) code of good practice for civil society participation in decision-making recommends involvement in all these steps of the decision-making process. This level of participation requires resources and training to build the capacity of CSOs to be equal players in the process. As outlined in the workshop, the informal support and mentoring by key allies in state agencies and more experienced CSOs was found to be crucial in building the capacity of advocates to move into the realm of policy advocacy and formal politics. Here, the importance of networks and relationship building—key aspects of CSO’s work—helped to facilitate their entry and performance in complex systems with multiple agendas.

However, formal access to resources (such as funded secretariats and peer navigators) is vitally important to assist CSOs in negotiating their way through public institutions and multi-lateral bodies, and to develop the high-level advocacy skills needed to influence policy and formal political institutions. Resources to enable payment for travel, subsistence, childcare and time preparing and attending meetings, similar to how state agents are paid, should be made available also.

Similarly, there is a need for formal structures to facilitate CS participation – not just ad hoc meetings as need arises. CSI needs to be structured and deliberate. This extends to practical matters such as the circulation of minutes, the ability to schedule / recommend agenda items, formal terms of reference for groups and so on.

The legitimacy of CSOs to act on behalf of their constituents (whether peers, service users, the public) is often queried. To meet this challenge, CSOs have established networks and coalitions and strive to ensure these networks are transparent and open, and have clear terms of reference. Formalising structures (such as, becoming an incorporated company/charity) can help in this, but can be a challenge in and of itself. CSOs that developed representative systems found it frustrating when state agencies and international institutions bypassed these systems and undermined the legitimacy of the network.

Building trust and rapport with policy makers is another challenge. Being a formal organisation or a network can help, so too can CSO′s advocacy style. Delivering arguments in an objective way that is familiar to policy makers—grounded in evidence and focused on fact— may help build rapport. Acknowledging that it is the state and its agencies that are the final arbiter of policy implementation, not the CSO, may help improve working relationships.

Embedding and institutionalising CSO participation in policy making can be a challenge. For example, CSOs may be included in one aspect of policy (e.g. policy formation), but not in another aspect of the same policy (e.g. implementation). CSOs seek to be included in all stages of policy development from agenda setting to monitoring and evaluating. Ensuring that policy is developed and delivered on a true partnership basis with shared responsibility for success and failure can help ensure participation goes across the entire policy process.

## Conclusion

Harm reduction internationally is facing new developments and challenges. The provision of harm reduction services is inadequate in many countries, and abstinence models receive increased support even in counties where harm reduction has been strong traditionally. In times of public health crises for people who use drugs (such as with the onset of HIV and AIDS in the 1980s; increasing overdose deaths in 2000s; and COVID-19 currently), CSOs have demonstrated their capacity to take the initiative, and to lead, act, and be bold in addressing the harm reduction needs of their communities. Drug user groups and service provider CSOs are able to access the people and places that state services are unable or reluctant to either because they are constrained by risk management or abstentionist ideologies. As the COVID-19 pandemic began to surge globally in 2020, the best harm reduction practices have emerged from CSOs and state agencies working in partnership—one providing the resources, the other the grassroots knowledge of how and where to act. Operationalising this partnership on an ongoing basis requires mutual trust and recognition of the strengths and limitations of both sectors, and a mutual desire to reduce the harms for people who use drugs. The lessons drawn from our workshop will help inform all partners on this pathway.

## Data Availability

Data sharing not applicable to this article as no datasets were generated or analysed during the current study.

## Footnotes

1 By civil society organisations, we mean non-governmental, voluntary and community organisations that operate in the associational space between the state and market where people associate to advance common interests [5].

2 The GIPA (Greater Involvement of People Living with AIDS) principles provide a clear commitment from the UN and governments that informs the planning of meetings, consultations, the development of guidelines, and policy development.

3 The Pompidou Group is the Council of Europe’s core mission is to contribute to the development of multidisciplinary, innovative, effective and evidence-based drug policies in its Member States. It seeks to link Policy, Practice and Science and focuses especially on the realities of local implementation of drug programmes. https://www.coe.int/en/web/pompidou/home.
